# Investigation of Infectious Disease Risks Associated With a Nontransplant Anatomical Donation Center — Arizona, 2014

**Published:** 2014-05-02

**Authors:** Marie A. de Perio, Bruce P. Bernard, Lisa J. Delaney, Nicki Pesik, Nicole J. Cohen

**Affiliations:** 1 National Institute for Occupational Safety and Health, CDC; 2 National Center for Emerging and Zoonotic Infectious Diseases, CDC

CDC is investigating reports of potential occupational exposure to human immunodeficiency virus (HIV), hepatitis B virus (HBV), hepatitis C virus (HCV), and *Mycobacterium tuberculosis* among workers performing preparation and dissection procedures on human nontransplant anatomical materials at a nontransplant anatomical donation center in Arizona. CDC is working with Arizona public health officials to inform persons exposed to these potentially infected materials. Nontransplant anatomical centers around the United States process thousands of donated cadavers annually. These materials (which might be fresh, frozen, or chemically preserved) are used by universities and surgical instrument and pharmaceutical companies for medical education and research. The American Association of Tissue Banks has developed accreditation policies for nontransplant anatomical donation organizations (1). It also has written standards (1) that specify exclusion criteria for donor material, as well as use of proper environmental controls and safe work practices to prevent transmission of infectious agents during receipt and handling of nontransplant anatomical materials. At the center under investigation, which is now closed, these standards might not have been consistently implemented.

CDC has assisted Arizona public health officials in notifying former workers at the center regarding potential exposure to HIV, HBV, and HCV, and *M. tuberculosis* while preparing nontransplant anatomical materials. Bloodborne pathogens (e.g., HIV, HBV, and HCV) can be transmitted when blood or other potentially infectious materials contact mucous membranes, such as the eyes, mouth, or nonintact skin, or when they enter the body through a percutaneous injury such as a needlestick or scalpel wound. *M. tuberculosis* can be transmitted by infectious aerosols generated by manipulation of infectious tissues.

Arizona public health officials have offered former workers at the center cost-free testing for HIV, HBV, and HCV, and *M. tuberculosis* infection as well as counseling regarding these infections. End users of nontransplant anatomical materials for medical training or research purposes are thought to be at considerably lower risk for infection because of the reduced survival and infectivity of these organisms over time, and are being notified separately where possible. Waste treatment, storage, and transportation workers handling containers or packaged nontransplant anatomical materials would not directly contact these materials during regular work and are not considered to be at risk unless there is a spill of infectious material. If a spill were to occur, proper disinfection procedures, determination of employee exposure, and worker follow-up with an assessment of transmission risk should take place, per facility protocols and the Bloodborne Pathogens Standard of the Occupational Safety and Health Administration (OSHA) (2). There are no known risks to the general public, and these activities are unrelated to organs or tissues recovered for transplantation in human recipients.

Employers and employees in the nontransplant anatomical donation industry and end users should recognize that cadavers and nontransplant anatomical materials are considered potentially infectious with *M. tuberculosis* and other pathogens, even if they are known to test negative for HIV, HBV, and HCV. Employers must comply with the OSHA Bloodborne Pathogens Standard, which requires a written exposure control plan, use of engineering and work practice controls, appropriate personal protective equipment, and provision of hepatitis B vaccine to employees assigned to jobs with occupational exposure risk (2). CDC’s Guidelines for Safe Work Practices in Human and Animal Medical Diagnostic Laboratories (3) and the Standards for Non-Transplant Anatomical Donation have been published (1). When transported, these materials should be packaged and labeled in accordance with all applicable regulations. Should a spill or damage to a package of nontransplant anatomical materials occur, procedures, such as those found in the U.S. Postal Service, Handbook EL–812, Hazardous Materials and Spill Response (4) and U.S. Department of Transportation Hazardous Materials Regulations (5) should be followed.
